# Does altitude level of a prior time‐trial modify subsequent exercise performance in hypoxia and associated neuromuscular responses?

**DOI:** 10.14814/phy2.12804

**Published:** 2016-07-19

**Authors:** Olivier Girard, Simone Bula, Raphaël Faiss, Franck Brocherie, Guillaume Y. Millet, Grégoire P. Millet

**Affiliations:** ^1^ Department of PhysiologyFaculty of Biology and MedicineISSULInstitute of Sport SciencesUniversity of LausanneLausanneSwitzerland; ^2^ Human Performance LaboratoryFaculty of KinesiologyUniversity of CalgaryCalgaryABCanada

**Keywords:** Altitude, cycling time‐trial, hypoxia severity, neuromuscular fatigue, post‐exercise recovery

## Abstract

We examined the influence of prior time‐trials performed at different altitudes on subsequent exercise in moderate hypoxia and associated cardiometabolic and neuromuscular responses. In normobaric hypoxia (simulated altitude 2000 m; FiO_2_: 0.163), 10 healthy males performed (1) an incremental test to exhaustion (VO_2max_2000_) and (2) a test to exhaustion at 80% of the power output associated to VO_2max_2000_ for a reference time (947 ± 336 sec). Thereafter, two sessions were conducted in a randomized order: a cycle time‐trial corresponding to the reference time (TT_1_) followed 22 min later (passive rest at 2000 m) by a 6‐min cycle time‐trial (TT_2_). TT_1_ was either performed at 2000 or 3500 m (FiO_2_: 0.135), while TT_2_ was always performed at 2000 m. As expected, during TT_1_, the mean power output (247 ± 42 vs. 227 ± 37 W; *P* < 0.001) was higher at 2000 than 3500 m. During TT_2_, the mean power output (256 ± 42 vs. 252 ± 36 W) did not differ between conditions. Before and after TT_1_, maximal isometric voluntary contraction torque in knee extensors (pooled conditions: −7.9 ± 8.4%; *P* < 0.01), voluntary activation (−4.1 ± 3.1%; *P* < 0.05), and indices of muscle contractility (peak twitch torque: −39.1 ± 11.9%; doublet torques at 100 Hz: −15.4 ± 8.9%; 10/100 Hz ratio: −25.8 ± 7.7%; all *P* < 0.001) were equally reduced at 2000 m or 3500 m. Irrespective of the altitude of TT_1_, neuromuscular function remained similarly depressed after TT_1_ both before and after TT_2_ at 2000 m. A prior time‐trial performed at different altitude influenced to the same extent performance and associated cardiometabolic and neuromuscular responses during a subsequent exercise in moderate hypoxia.

## Introduction

Fatigue is a disabling symptom in which physical and cognitive function is limited by interactions between performance fatigability (i.e., the decline in an objective measure of performance such as contractile function and/or muscle activation) and perceived fatigability (i.e., sensations that regulate the integrity of the performer) (Enoka and Duchateau 2016). Under this frame, prior exercise that challenges multiple physiological (i.e., cardiorespiratory, metabolic, neuromuscular) regulatory systems and thereby modifies effort perception and influences performance during the completion of a subsequent exercise bout (Karlsson et al. 1975; Hogan and Welch 1984; Amann 2011). Currently, there is renewed interest in the methodological approach that consists in manipulating the amount/severity/type of pre‐established fatigue levels through the induction of localized fatigue of specific muscle groups (e.g., neuromuscular electrical stimulation protocol of the quadriceps; Hureau et al. 2014) or following the completion of an initial whole‐body exercise bout (Amann and Dempsey 2008; Girard et al. 2015).

In one study, Amann and Dempsey (2008) demonstrated that the induction of different levels of pre‐existing locomotor muscle fatigue [following the completion of constant‐load cycling of ~10 min at ~347 W (83% of peak power output) vs. ~276 W (67%)] had a substantial dose‐dependent influence (−6%) on performance time during a subsequent (i.e., 4 min later) 5‐km time‐trial (TT). Specifically, the higher the level of pre‐existing locomotor muscle fatigue, as assessed via pre‐ and post‐exercise magnetic femoral nerve stimulation, the lower the central motor drive and power output during the subsequent TT. A striking finding, however, was that the end of the subsequent TTs coincided with an almost identical level of peripheral fatigue, independent of the level of pre‐existing fatigue and/or the marked differences in exercise performance. Authors claimed that feedbacks from fatiguing muscles play an important role in the determination of central motor drive and force output, and that the development of peripheral muscle fatigue is confined to a certain level (also referred as a “critical” threshold), so as not to surpass a sensory tolerance limit.

Increasing the degree of environmental stress such as the ambient hypoxia severity [i.e., a reduction in environmental oxygen (O_2_) availability] is known to exacerbate exercise‐induced demands (and thereby recovery requirements) in turn leading to excessive fatigue levels (Amann 2011). With this in mind, we recently manipulated hypoxia severity during an initial set of repeated sprints (eight 5‐sec sprints; 30 sec rest) under normoxia, moderate, or severe hypoxia and examined the effects on performance and lower limbs neuromuscular activity during a subsequent set (four sprints) performed 6 min later in normoxia (Girard et al. 2015). Despite sprint performance and neural alterations were largely influenced by the hypoxia severity in the initial set, hypoxia had no residual effect during the subsequent normoxic set (i.e., similar fatigue pattern across conditions), yet the absence of negative “carry‐over” effects would still need to be confirmed under hypoxic conditions. In addition, central and peripheral mechanisms underpinning neuromuscular fatigue, likely influenced by hypoxia severity, were not assessed in this latter study.

To date, the majority of studies investigating the interplay between central and peripheral mechanisms of fatigue through hypoxic perturbations have employed exhaustive, whole‐body continuous exercise (Amann et al. 2006a) and repeated, brief submaximal (Millet et al. 2009, 2012; Goodall et al. 2010) or maximal (Christian et al. 2014) isometric contractions. In comparison to exercising at fixed work rate to fatigue (i.e., open‐loop design), self‐paced exercise (i.e., time‐trial) of predetermined duration/distance/work (i.e., closed‐loop designs) involves pacing strategies for achieving optimal performance. It well described that TT performance and associated physiological responses vary between hypoxic and normoxic conditions (Amann et al. 2006b; Périard and Racinais 2016; Saugy et al. 2016). The extent to which the neuromuscular consequences also differ between normoxic and a range of moderate‐to‐severe hypoxic conditions is not clear.

Our intention was therefore to examine the influence of prior TT performed at different altitudes on subsequent exercise performance in moderate hypoxia and associated cardiometabolic and neuromuscular responses. We anticipated that severer hypoxia during a prior TT would exaggerate the demands placed on various physiological regulatory systems, in turn resulting in lower power output. We were unsure, however, whether specific recovery requirements and fatigue‐related residual or “carry‐over” effects would differently influence performance and associated cardiometabolic and neuromuscular responses during a subsequent exercise performed in same moderate hypoxia.

## Methods

### Participants

Ten healthy men (Mean ± SD: age 34.4 ± 6.8 years; height 180.4 ± 7.7 cm; body weight 78.6 ± 10.9 kg; body fat 15.0 ± 5.9%) volunteered to participate in the study. All participants were born and raised at <1000 m and had not traveled to elevations >1000 m in the 3 months prior to investigation. They gave their informed, written consent preceding the commencement of the experiment. Experimental protocol was conducted according to the Declaration of Helsinki for use of Human Subjects and approved by the Ethics Committee of Valais, Switzerland (CCVEM 007/10).

### Experimental design

Participants visited the laboratory on two occasions, separated by 3–7 days, before the two main experimental trials. During the first preliminary visit, they were initially familiarized with neuromuscular testing and performed an incremental test in normobaric hypoxia at a simulated altitude of 2000 m (FiO_2_ 0.163) for maximal oxygen uptake determination (VO_2max_2000_). During a subsequent preliminary visit, again at 2000 m, participants cycled at constant workload (80% of the power output associated with their VO_2max_2000_: 245 ± 42 W) to exhaustion (Tlim = 947 ± 336 sec). Exercise was terminated when pedaling rate dropped below 60 rpm for >5 sec (exhaustion). Thereafter, two sessions were conducted in a randomized order: a cycle time‐trial (TT_1_) with Tlim as individualized reference duration followed 22 min later (passive rest at 2000 m) by a 6‐min cycle time‐trial (TT_2_). TT_1_ was either performed at 2000 or 3500 m (FiO_2_ 0.135), while TT_2_ was always performed at 2000 m. After preliminary tests, the duration of 22 min between the two time‐trials was chosen as a good trade‐off between the times needed to allow significant perceptual recovery from TT_1_, with the parallel methodological requirement to keep recovery time short enough for a partial recovery of neuromuscular function, both likely to influence subsequent efforts (Minett and Duffield 2014).

### Testing procedures

Testing was conducted in a normobaric hypoxic chamber (SL ‐ 400, ATS, Sydney, Australia) of ~30 m^3^ (2.4 m × 5.0 m × 2.5 m) maintained at a constant temperature of ~25°C and ~40% relative humidity. Prior to each cycling bout, FiO_2_ (Oximeter Gox 100, Greisinger, Germany) as well as room temperature (°C) and humidity (%) were measured. This chamber allowed modifying the simulated altitude between 3500 and 2000 m in less than 5 min. All tests were performed on a computer‐controlled electrically braked cycle ergometer (Lode Excalibur Sport, Groningen, the Netherlands). Participants performed their trials at the same time of the day (±1 h) and wore similar sports gear (cycling shoes, short and jersey). They were instructed to refrain from any strenuous physical activity and maintain their normal diet (i.e., avoiding any nutritional supplements, caffeine, or alcohol consumption) and sleeping habits (≥7 h/night) for the 24 h before each test. They were encouraged to drink 4–6 mL of water per kilogram of body mass every 2.5 h on the day before each experimental session to ensure euhydration at the start of exercise.

#### Preliminary sessions

During the first preliminary session, participants’ anthropometrical parameters (body height, body weight) and body composition (Bod Pod, Cosmed US Inc., Concord) were first measured. Then, they were requested to perform maximal voluntary contractions (MVC) of the knee extensors until they felt accustomed to the equipment; the coefficient of variation in three successive trials was <5%. Afterwards, the optimal stimulation intensity for one single stimulus was determined by increasing the current gradually from 10 mA until there was no further increase in peak twitch torque and concomitant maximal M‐wave amplitudes. This intensity was further increased by 30% (e.g., supramaximal) and subsequently maintained for the entire session. Thereafter, participants performed the complete procedure of neuromuscular tests (see below; total duration ~3 min). Then, they entered the normobaric hypoxic chamber at a simulated altitude of 2000 m, and after ~30 min rest, the incremental test was undertaken. The protocol consisted of cycling for 5‐min warm‐up at 60 W, then at a starting power output of 90 W and increasing every minute by 30 W until volitional exhaustion, despite strong verbal encouragement. The highest 30 sec average value of VO_2_ (see below) was defined as VO_2max_2000._

The second preliminary session was conducted inside the hypoxic chamber at a simulated altitude of 2000 m as follows: (1) rest in a seated position for 30 min; (2) Participants cycled at constant workload (80% of the power output associated with their VO_2max_2000_) to exhaustion.

#### Experimental sessions

Each of the two main experimental session were conducted as follows: (1) rest in a seated position for 30 min inside the chamber, while participants were instrumented; (2) a 10 min cycle warm‐up at 60 W (pedaling rate 70–80 rpm); (3) neuromuscular tests before TT_1_ (pre‐TT_1_); (4) 5‐min rest; (5) cycle time‐trial for Tlim duration (TT_1_); (6) 22‐min rest at 2000 m including neuromuscular tests ~1 min after TT_1_ (post‐TT_1_) and ~2 min before TT_2_ (pre‐TT_2_); (7) 6‐min cycle time‐trial (TT_2_); (8) neuromuscular tests ~1 min after TT_2_ (post‐TT_2_).

#### Cycling time‐trials

During each time‐trial, participants were asked to maintain the highest sustainable effort, while receiving strong verbal encouragements. The starting work rate was 80% of individual power output at VO_2max_2000_ for all exercise trials. Participants were informed of every minute elapsed during time‐trials. They were able to continuously self‐regulated power output (±10 W). This research was run in double‐blinded, controlled manner. Participants were told that the overall goal of the experiment was to test the reproducibility of their cycling time‐trial performance in hypoxia, yet without any accurate information about the randomized simulated altitude levels inside the hypoxic room that were set and controlled by an independent research assistant.

#### Neuromuscular function

The neuromuscular assessment consisted of a 4‐sec MVC of the knee extensors with a superimposed 100 Hz doublet (Db100) applied to the peripheral motor nerve when torque had reached a visible plateau. This was followed after 3 sec by (1) one Db10, (2) one Db100, and (3) three single twitches in a relaxed state (all separated by 3 sec). This neuromuscular testing was conducted three times, while ~60 sec of passive rest separated each MVC. Prior to the pre‐TT_1_ neuromuscular assessment, participants were warmed up by completing 5 × 4‐sec MVC with progressively increasing subjective effort (starting at 50% of subjective maximal effort with increments of 10%) followed by 2 × 4‐sec MVC (separated by 1 min of passive rest).

### Measurements

#### Cardiopulmonary and respiratory responses

Heart rate (HR), monitored via a wireless Polar monitoring system (Polar Electro Oy, Kempele, Finland) and pulse oxygen saturation (Spo_2_), estimated noninvasively via pulse oximetry using an earlobe probe (Nonin, Wristwatch, McAllen), were recorded immediately prior to entering the chamber and every 60 sec during exercise. Rating of perceived exertion (RPE) was obtained using the 6–20 Borg scale. The following respiratory variables and pulmonary gas exchange parameters were measured breath‐by‐breath at rest and throughout all exercises using the portable analyzer Metamax 3b (Cortex Biophysik, Leipzig, Germany). The following parameters were measured: oxygen uptake [VO_2_ (mL kg^−1^ min^−1^)], minute ventilation [VE (L min^−1^)], carbon dioxide production [VCO_2_ (L min^−1^)], respiratory exchange ratio (RER), ventilatory equivalent for O_2_ (VE/VO_2_), and breathing frequency [BF (breath min^−1^)]. Finally, a capillary blood sample was taken from the fingertip and analyzed for blood lactate concentration with the Lactate Pro (LT‐1710, Arkray, Japan) portable analyzer, 2 min before and exactly 2 min after TT_1_ and TT_2_.

#### Force and electromyographic recordings

During all neuromuscular assessments, participants were seated upright on a custom‐built adjustable chair with the hips and knees flexed at 90° (0° corresponding to full knee extension). Restraining straps placed across the chest and hips secured the participants in the chair to prevent extraneous movement, while the dynamometer (Captels, St Mathieu de Treviers, France) was attached 3–5 cm above the tip of the lateral malleoli.

Electromyographic (EMG) signals of the VL and *rectus femoris* (RF) muscles (cycling and neuromuscular assessment) were recorded via bipolar Ag/AgCl electrodes (Ambu Blue sensor T; Ambu A/S, Denmark) with a diameter of 9 mm and an interelectrode distance of 25 mm. Before electrode placement, the skin was lightly abraded and washed to remove surface layers of dead skin, hair, and oil. The ground electrode was attached to the right wrist. The position of the EMG electrodes was marked with indelible ink to ensure that they were placed in the same location during subsequent trials. To ensure low levels of movement artifact, electrode cables were fastened to participant’ bodies with medical adhesive tape and wrapped in net. The myoelectric signal was amplified (Octal Bioamp, ML138, ADInstruments, Oxfordshire, UK; input impedance = 200MΩ, common mode rejection ratio >96 dB, gain = 1000), band‐pass filtered (bandwidth frequency = 5 to 500 Hz), digitized (sampling frequency = 2,000 Hz), acquired, and later analyzed (LabChart v7.0, ADInstruments Inc, Oxfordshire, UK) with force signal.

#### Femoral nerve stimulation

A high‐voltage (maximal voltage 400 V) constant current stimulator (Digitimer DS7AH; Digitimer, Hertfordshire, UK) was used to deliver square‐wave stimuli of 1 ms duration. The femoral nerve was stimulated percutaneously via a 10 mm diameter self‐adhesive cathode electrode (Skintact, Austria) pressed manually by the investigator onto the skin at the femoral triangle. The anode, a self‐adhesive pad (5 × 10 cm, Medicompex; Ecublens, Switzerland) was applied to the gluteal fold.

### Data analysis

Power output, cardiorespiratory and pulmonary as well as EMG data were averaged over each entire cycling bout to obtain one value for TT_1_ (TT_1_2000_ vs. TT_1_3500_) and TT_2_ (TT_2_2000_ vs. TT_2_3500_)

For each neuromuscular test sequence, voluntary force (MVC force) was recorded over the highest 1‐sec plateau preceding the superimposed twitch. The peak potentiated twitch force (i.e., the highest value of twitch tension production, Pt) was determined from the mechanical response of the three evoked twitches (and averaged to obtain one Pt value) and one paired doublet at 10 Hz and 100 Hz (Db10 and Db100, respectively). The peak‐to‐peak amplitude of the concomitant VL and RF M‐waves during the three resting twitches was measured and averaged across the three stimulations to obtain one representative M‐wave value. Voluntary activation (VA) was assessed using twitch interpolation and defined as follows: VA (%) = {[1 – (superimposed Db100 doublet/resting potentiated Db100)] × 100}. From the mechanical response induced by paired high‐frequency [100 Hz (i.e., 10‐ms interstimulus interval)] and low‐frequency [10 Hz (i.e., 100 ms interstimulus interval)] supramaximal electrical stimulation, the low‐ to high‐frequency torque ratio was calculated (10/100 Hz) and used as a surrogate of low‐ and high‐frequency tetanic stimulations (Vergès et al. 2009). For all the neuromuscular parameters, the values of three trials were averaged for subsequent analysis. The reliability of measurements of central and peripheral fatigue specific to the quadriceps both before and after “fatigue” has been reported elsewhere (Place et al. 2007).

### Statistics

Values are expressed as mean ± SD. For TT_1_ and TT_2_ separately, paired samples *t*‐tests were used to compare cardiorespiratory and pulmonary and EMG data. Two‐way analysis of variance (ANOVAs) [Time (pretests and post‐tests) × Condition (TT_1_ and TT_2_)] were also used to compare neuromuscular responses. To assess the assumptions of variance, Mauchly's test of sphericity was performed using all ANOVA results. A Greenhouse‐Geisser correction was performed to adjust the degree of freedom if an assumption was violated, while post hoc pairwise comparisons with Bonferroni‐adjusted *P* values were performed if a significant main effect was observed. For each ANOVA, partial eta‐squared was calculated as measures of effect size. Values of .01, .06, and above .14 were considered as small, medium, and large, respectively. All statistical calculations were performed using SPSS statistical software V.21.0 (IBM Corp., Armonk, NY). The significance level was set at *P* < 0.05.

## Results

### Preliminary sessions

At a simulated altitude of 2000 m, the maximal oxygen uptake (VO_2max_2000_) was 54.9 ± 7.3 mL kg^−1^ min^−1^ and peak power output was 306 ± 53 W. Tlim performed at 245 ± 42 W was 947 ± 336 sec.

### Prior time‐trial (TT_1_)

**Figure 1:**
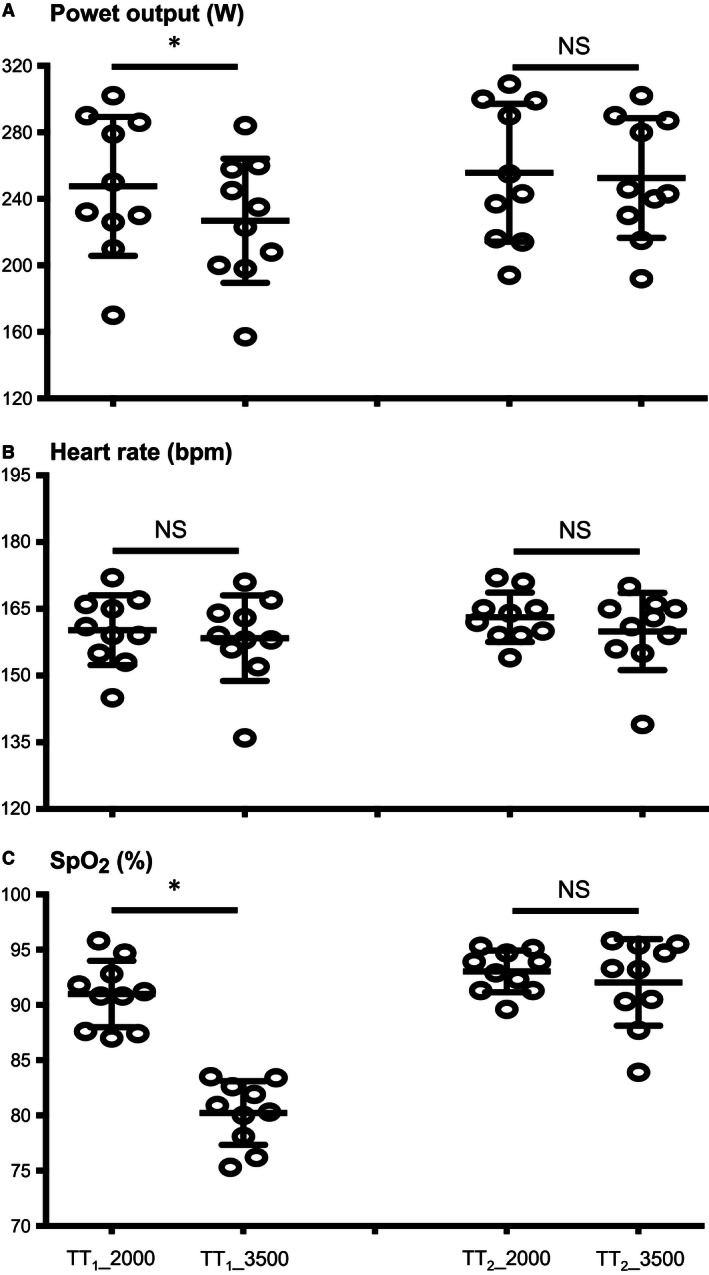
Averaged power output (A), heart rate (B) and pulse oxygen saturation (C, Spo_2_) during the first (TT_1_) and during the second (TT_1_) cycling bouts. The first cycling bout (TT_1_) was performed at 2000 m or 3500 m, while the second bout (TT_2_) was always performed at 2000 m. Mean ± SD (*n* = 10). *Denotes a significant difference between 2000 m and 3500 m (*P* < 0.05).

Mean power output (247 ± 42 vs. 227 ± 37 W; +8.2 ± 3.6%; *P* < 0.001) and Spo_2_ (+11.8 ± 1.9%; *P* < 0.001) were significantly higher during TT_1_2000_ versus TT_1_3500_ (Fig. 1). Although VO_2_ (+8.9 ± 7.8%; *P* < 0.01) was higher during TT_1_2000_ in reference to TT_1_3500_, RER (−6.1 ± 6.7%; *P* < 0.05), VE/VO_2_ (−19.9 ± 8.8%; *P* < 0.001), and BF (−10.8 ± 12.1%; *P* < 0.05) were lower (Table 1). Averaged RMS activity both for VL and RF muscles did not differ between TT_1_2000_ and TT_1_3500_ (Table 1). RPE values measured at the completion of TT_1_ did not differ (*P* > 0.05) between TT_1_2000_ (17.0 ± 1.5) and TT_1_3500_ (17.0 ± 1.2).

**Table 1:** Average responses to exercise during the first cycling bout at 2000 m (TT_1_2000_) and 3500 m (TT_1_3500_)

	TT_1_2000_	TT_1_3500_	ANOVA *P* value
Cardiopulmonary variables			
HR (bpm)	160 ± 8	158 ± 10	*P* = 0.147
Spo_2_ (%)	91.0 ± 3.0	80.2 ± 3.1	***P*** ** < 0.001**
VO_2_ (mL min^−1 ^kg^−1^)	44.0 ± 7.3	39.9 ± 5.8	***P*** ** = 0.005**
VE (L min^−1^)	115 ± 22	124 ± 19	*P* = 0.079
RER	0.95 ± 0.04	1.01 ± 0.08	***P*** ** = 0.024**
VE/VO_2_	32.3 ± 4.1	38.6 ± 4.0	***P*** ** < 0.001**
BF (breaths min^−1^)	40 ± 5	44 ± 5	***P*** ** = 0.014**
[La] (mmol L^−1^)	10.2 ± 2.5	10.7 ± 3.1	*P* = 0.597
Surface EMG activity			
RMS VL (mV)	0.165 ± 0.062	0.159 ± 0.058	*P* = 0.547
RMS RF (mV)	0.069 ± 0.027	0.066 ± 0.023	*P* = 0.435

Mean ± SD (*n* = 10). HR, heart rate, Spo_2_; arterial oxygen saturation; VO_2_, oxygen consumption; VE, minute ventilation; RER, respiratory exchange ratio; BF, breathing frequency; [La], blood lactate concentration; RMS VL and RF, root mean square of vastus lateralis and rectus femoris muscles. Bold values indicate the significant of *P* ≤ 0.05.

### Subsequent time‐trial (TT_2_)

Mean power output (256 ± 42 vs. 252 ± 36 W; +0.9 ± 4.1%; *P* > 0.05) along with accompanying cardiopulmonary and quadriceps muscle activation responses did not differ between TT_2_2000_ and TT_2_3500_ (Fig. 1; Table 2). RPE values measured at the completion of TT_2_ did not differ (*P* > 0.05) between TT_2_2000_ (16.9 ± 1.5) and TT_2_3500_ (16.3 ± 1.7).

**Table 2:** Average responses to exercise during the second cycling bout after completing the first time‐trial at 2000 m (TT_2_2000_) and 3500 m (TT_2_3500_)

	TT_2_2000_	TT_2_3500_	ANOVA *P* value
Cardiopulmonary variables			
HR (bpm)	163 ± 6	160 ± 9	*P *=* *0.101
Spo_2_ (%)	93.0 ± 1.9	91.7 ± 4.0	*P *=* *0.188
VO_2_ (mL min^−1^ kg^−1^)	43.6 ± 7.9	42.7 ± 6.8	*P *=* *0.957
VE (L min^−1^)	116 ± 22	116 ± 14	*P *=* *0.751
RER	0.78 ± 0.02	0.80 ± 0.03	*P *=* *0.361
VE/VO_2_	34.3 ± 4.0	33.4 ± 2.3	*P *=* *0.364
Fb (breaths min^−1^)	45 ± 7	43 ± 5	*P *=* *0.564
[La] (mmol L^−1^)	10.4 ± 2.2	10.4 ± 3.4	*P *=* *0.601
Surface EMG activity			
RMS VL (mV)	0.159 ± 0.049	0.168 ± 0.053	*P *=* *0.214
RMS RF (mV)	0.069 ± 0.029	0.071 ± 0.026	*P *=* *0.684

Mean ± SD (*n* = 10). HR, heart rate, Spo_2_; arterial oxygen saturation; VO_2_, oxygen consumption; VE, minute ventilation; RER, respiratory exchange ratio; Fb, breathing frequency; [La], blood lactate concentration; RMS VL and RF, root mean square of vastus lateralis and rectus femoris muscle.

### Neuromuscular consequences

**Figure 2:**
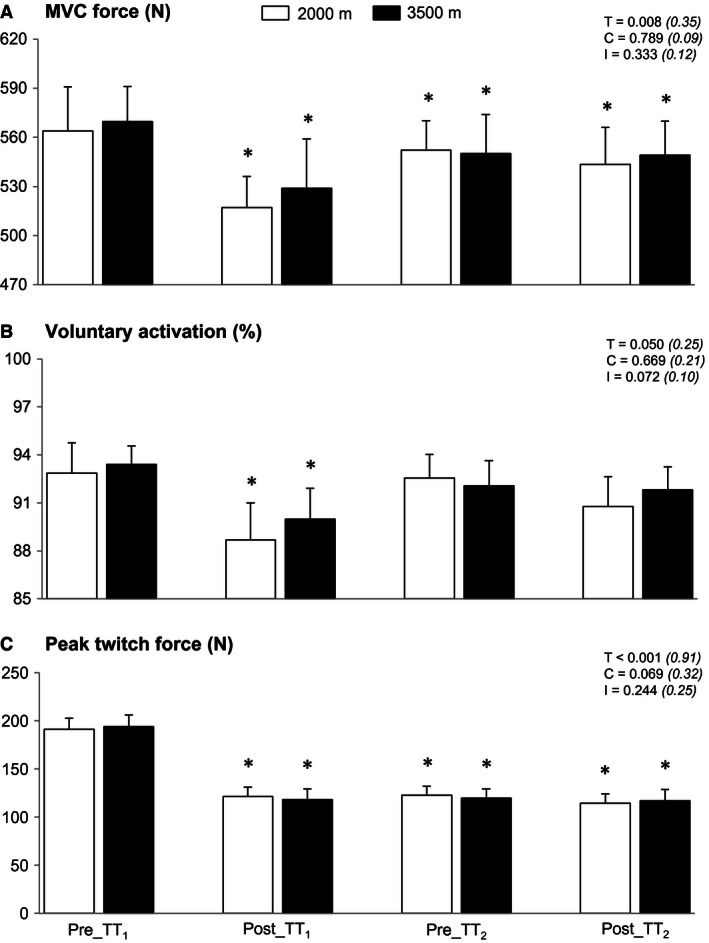
Maximal voluntary contraction force (A), voluntary activation (B), and peak twitch force (C) before and after the first and the second cycling bouts. The first cycling bout (TT_1_) was performed at 2000 m or 3500 m, while the second bout (TT_2_) was always performed at 2000 m. Mean ± SD (*n* = 10). *Denotes a significant difference from pre‐TT_1_ (*P* < 0.05).

Maximal voluntary contractions torque (pooled conditions: −7.9 ± 8.4%; *P* < 0.01), voluntary activation (−4.1 ± 3.1%; *P* < 0.05), and indices of muscle contractility (peak twitch torque: −39.1 ± 11.9%; doublet torques at 10 Hz and 100 Hz: −38.7 ± 10.2% and −15.4 ± 8.9%; 10/100 Hz ratio: −25.8 ± 7.7%; all *P* < 0.001) were equally reduced from pre‐TT_1_ to post‐TT_1_, whereas M‐wave characteristics of both VL and RF muscles did not differ (Fig. 2; Table 3). Compared to post‐TT_1_, MVC force and indices of muscle contractility remained similarly depressed at pre‐TT_2_ and did not further decrease at post‐TT_2_ time points. Similarly, voluntary activation values returned to baseline levels at pre‐TT_2_ and remained unchanged post‐TT_2_. For this parameter, no differences were found between conditions either.

**Table 3:** Muscle function before and after the first (TT_1_) and the second cycling bout (TT_2_)

Variables	Condition	Time points				ANOVA *P* value (ES)		
		Pre‐TT_1_	Post‐TT_1_	Pre‐TT_2_	Post‐TT_2_	Time	Condition	Interaction
Db10 (*N*)	2000 m	305 ± 51	192 ± 49^a^	195 ± 41^a^	189 ± 51^a^	<0.001	0.087	0.282
	3500 m	319 ± 53	203 ± 50^a^	205 ± 41^a^	191 ± 46^a^	(0.93)	(0.29)	(0.13)
Db100 (*N*)	2000 m	289 ± 41	242 ± 50^a^	244 ± 43^a^	247 ± 49^a^	<0.001	0.088	0.361
	3500 m	299 ± 40	256 ± 41^a^	250 ± 34^a^	250 ± 37^a^	(0.78)	(0.29)	(0.11)
Db10.Db100^−1^	2000 m	1.05 ± 0.08	0.79 ± 0.09^a^	0.80 ± 0.07^a^	0.76 ± 0.09^a^	<0.001	0.616	0.534
	3500 m	1.07 ± 0.06	0.79 ± 0.10^a^	0.81 ± 0.08^a^	0.76 ± 0.09^a^	(0.93)	(0.31)	(0.06)
M‐wave VL (mV)	2000 m	20.6 ± 4.5	20.8 ± 4.7	19.2 ± 4.0	19.6 ± 3.8	0.061	0.100	0.469
	3500 m	21.7 ± 5.2	22.1 ± 5.0	20.7 ± 4.2	20.4 ± 4.3	(0.30)	(0.27)	(0.07)
M‐wave RF (mV)	2000 m	8.6 ± 2.2	8.2 ± 2.1	7.9 ± 2.0	7.7 ± 1.9	0.442	0.822	0.776
	3500 m	8.7 ± 3.2	8.5 ± 3.1	8.2 ± 2.7	8.3 ± 2.9	(0.93)	(0.06)	(0.04)

Db10, force associated with doublet at 10 Hz; Db100, force associated with doublet at 100 Hz; VL, vastus lateralis; RF, rectus femoris.

a Denotes a significant difference from pre‐TT_1_ (*P* < 0.05).

## Discussion

### Summary of main findings

The novel findings were as follows: (1) Neuromuscular adjustments resulting from the completion of the initial cycling bout of same duration were independent of the hypoxia severity with, in particular, profound alterations in muscle contractility (i.e., low‐frequency fatigue); and (2) There was no influence of the altitude of a prior time‐trial on performance and associated cardiometabolic responses, with no additional muscle fatigue development, during a subsequent 6‐min time‐trial performed in moderate hypoxia.

We further demonstrated that performance is impaired during a time‐trial (approximately 16 min) conducted at a simulated altitude of 3500 m versus 2000 m, while active muscle (quadriceps) activation responses did not differ significantly. Also, after 22 min of passive rest, recovery of neuromuscular function was minimal in both conditions. Thus, we dispute the fact that altitude level of prior time‐trial influences performance and associated cardiorespiratory and neuromuscular responses during completion of a subsequent exercise of similar nature in moderate hypoxia.

### Neuromuscular fatigue characteristics post‐TT_1_ were not influenced by hypoxia severity

The development of neuromuscular fatigue (defined as a decrease in MVC force) from pre‐TT_1_ to post‐TT_1_ did not differ between the two levels of hypoxia. A substantial part of this fatigue was due to neural factors, consistent with previous time‐trial studies (Dahlstrom et al. 2013; Saugy et al. 2016). Hence, voluntary activation values dropped similarly by ~4%, signifying that muscle activation became suboptimal at the end of both TT_1_2000_ and TT_1_3500_. This was further accompanied by near‐identical level of peripheral muscle fatigue, whereas M‐wave amplitudes of VL and RF muscles were not affected by the time‐trial. This observation confirms that the measured changes in twitch amplitude are mainly due to changes occurring within the quadriceps and that neuromuscular propagation failure might be excluded. An increased rate of accumulation of metabolites (e.g., H+, Pi) can directly inhibit the contractile apparatus or disrupt the Ca^2+^ release and uptake pathways in the sarcoplasmic reticulum (Allen et al. 2008). A valid method to identify excitation–contraction coupling as a major factor of fatigue is the examination of force loss in response to low‐ (10 Hz) and high‐ (100 Hz) frequency paired stimuli (Vergès et al. 2009). As confirmed in this study, low‐frequency fatigue has already been identified as one of the main fatigue‐causing factor for high‐intensity exercises, irrespectively of hypoxia severity (Christian et al. 2014).

### No negative “carry‐over” effects on subsequent exercise in moderate hypoxia (TT_2_)

The duration of the recovery period after prior exercise (i.e., Spo_2_ recovery to initial values) may at least partially dictate performance during completion of a subsequent exercise bout. In this study, hypoxia severity during TT_1_ had no negative “carry‐over” (or residual) effect on performance fatigability and associated cardiorespiratory and EMG variables during TT_2_ completed after 22 min of rest in moderate hypoxia. In support, we recently observed that despite differing hypoxic severity levels (FiO_2_^ ^= 0.133, 0.168 and 0.209) during an initial set of eight 5‐sec sprints, performance and neuromechanical patterns did not differ during four additional sprints performed, 6 min later (passive rest), in normoxia (Girard et al. 2015). These results somehow contrasts with those of Amann et al. (2007) who indicated that after constant load cycling exercise to exhaustion in normoxia (~171 sec), moderate (~278 sec) and severe (~171 sec) hypoxia (FiO_2_ = 0.209, 0.15, and 0.10, respectively), hyperoxygenation via acute O_2_ supplementation (FiO_2_ = 0.30) caused participants to prolong exercise time at task failure in severe hypoxia (+171%), but not in normoxia and moderate hypoxia. Important methodological differences – that is, the range of Spo_2_ values for the more severe hypoxic conditions (67 vs. 80%), the nature of the exercise (constant load vs. time‐trial), the recovery duration between the two subsequent exercises (few seconds vs. 22 min), the O_2_ condition of the second exercise bout (hypoxia, normoxia vs. hyperoxia) – therefore only lead to anecdotal comparisons of performance and physiological responses between the aforementioned studies and the present one.

A further interesting observation was that the additional neuromuscular consequences of completing TT_2_ were minimal (i.e., no difference between pre‐TT_2_ and post‐TT_2_) in both conditions. The short duration (6 min) of the second time‐trial might partially explain why we failed to observe the further development of significant decrement in muscle activation (at least as for TT_1_), as greater central fatigue has been detected after longer time‐trials only (Thomas et al. 2015; Froyd et al. 2016); even though these observations have been obtained near sea level with no prior exercise. In this study, since the level of locomotor muscle fatigue pre‐TT_2_ versus post‐TT_2_ was also identical, the individual critical threshold of peripheral fatigue (Amann et al. 2006b) may have already been achieved when TT_2_ started. Despite exercise duration was approximately three times shorter, average power output maintained during TT_2_ resembles those produced during TT_1,_ with also similar power values between conditions.

### Prior time‐trial (TT_1_) performance was lower at severe versus moderate hypoxia

As expected, average power output was lower during TT_1_3500_ versus TT_1_2000_. Our results are in line with previous studies that showed that aerobic performance is altered with altitude: Compared to normoxia, this decrease was reported to be between 10 and 20% at 3000 m (Ventura et al. 2003) in normobaric hypoxia. In this study, we did not measure performance or VO_2max_ in normoxia and therefore cannot compare directly our data. Many studies (Peltonen et al. 2001; Wehrlin and Hallen 2006; Mollard et al. 2007; Périard and Racinais 2016) demonstrated that the reduced VO_2max_ was induced by a decrease in Spo_2_, leading to aerobic performance impairment. It is possible that the type of hypoxic condition (e.g., normobaric hypoxia) may explain the relatively moderate 8% difference in performance between the two TT_1_ since we recently showed that the performance is less altered in normobaric than hypobaric hypoxia (Saugy et al. 2016). The main mechanism for endurance performance impairment in hypoxia might be the decrease in VO_2max_ with a mean decrease of 7.7% per 1000 m increase in altitude [−4% at 1000 m (Wehrlin and Hallen 2006; Mollard et al. 2007); −10% (Mollard et al. 2007) to −15% (Peltonen et al. 2001; Wehrlin and Hallen 2006; Périard and Racinais 2016) at 2500–3000 m; or −30% (Mollard et al. 2007) at 4500 m, all in normobaric hypoxia]. This reduction in convective O_2_ transport during hypoxic cycling precipitate a decrement in peak exercise capacity and therefore a shift of a given absolute work load to a higher relative intensity.

Alterations in convective O_2_ transport to the working muscles are the result of changes in arterial oxygen content and/or limb blood flow (Romer et al. 2006). Obviously, the VO_2max_ was strongly linked to Spo_2_ levels also tainted by altitude (Chapman 2013). Reportedly, a > 3% reduction in Spo_2_ from rest has a significant detrimental effect on VO_2max_ (Harms et al. 1997). Spo_2_ maintenance, and not baseline VO_2max_ levels per se, is a primary limiting factor determining VO_2max_ decline with exposure to acute altitude (Chapman 2013). In this study, 2000 m and 3500 m simulated altitudes corresponded to Spo_2_ values of ~80 and ~90%, respectively, highlighting differences in the severity of hypoxic conditions. Hyperventilation of heavy sustained exercise (>85% VO_2max_) causes substantial increases in respiratory muscle work, leading to diaphragm and expiratory muscle fatigue (Dempsey et al. 2006) in turn reducing blood flow, and thus O_2_ delivery, to the working limb (Harms et al. 2000). To which extent this phenomenon would explain the observed lower performance during TT_1_3500_ compared to TT_1_2000_ could not be ascertained here.

In this study, TT_1_ was a performance test whereby the participants were continuously able to adjust their pace as they attempt to sustain the highest average power output for a predetermined reference time. Under more severe hypoxic conditions (>10% lower Spo_2_ values during TT_1_3500_ versus TT_1_2000_), reductions in power output occurred together with elevated cardiovascular load despite similar skeletal muscle recruitment (EMG). This probably ensured that the rate of peripheral fatigue development was slowed in the more severe hypoxic condition. To date, experimental data are contradictory, with some studies reporting no effect of severe hypoxia on EMG amplitude during dynamic muscle contractions (Donnelly and Green 2013), and other reporting increased activity with a muscle‐specific pattern (Fulco et al. 1996; Torres‐Peralta et al. 2014). During 5‐km cycle time‐trials, increased systemic O_2_ transport from hypoxia to hyperoxia (via wide changes in FiO_2_) resulted in parallel increases in central motor drive (skeletal muscle recruitment) and power output ultimately leading to improved time‐trial performance; yet, the magnitude of peripheral muscle fatigue developed at end‐exercise was identical (Amann et al. 2006b). In this study, perceived fatigability was not different between conditions, as evidenced by similar RPE values between TT_1_2000_ and TT_1_3500_.

### Post‐exercise recovery of neuromuscular function

Limited research is available to show how long a diminished neuromuscular response to intense cycling exercise will last, notably after hypoxic tasks. In this study, impairment in muscle function persisted at least 22 min into recovery, with remarkably similar post‐TT_1_ and pre‐TT_2_ peak twitch values. Although the extent to which muscle force decreased in response to TT_1_ varied according to the stimulation frequency considered (1 and 10 Hz vs. 100 Hz), at all three stimulation frequencies, there was a lack of recovery between post‐TT_1_ and pre‐TT_2_ time points. Contrastingly, VA values returned near baseline at pre‐TT_2_. Such apparent disconnect in time‐course changes in peripheral and central factors contributing to the ensuing (partial) recovery of neuromuscular performance (MVC force) before the completion of TT_2_ reinforces the contribution of the brain to performance recovery after strenuous exercises (Minett and Duffield 2014).

### Additional considerations/Limitations

One major flaw of almost all whole‐body exercise studies, including the present one, is that we did not measure fatigue during exercise per se nor right at exercise cessation since a short delay (~1 min) was needed to install the participants on the ergometer before testing. Although no statistically significant differences were found in MVC and VA values between mild hypoxic (FiO_2 _= 0.15) and normoxic (FiO_2 _= 0.209) environments, Dahlstrom et al. (2013) made the interesting observation that VA values collected at 1, 2, 3, and 4 min post‐20‐km cycle time‐trial were all significantly reduced compared to baseline. Contrastingly, substantial recovery in skeletal muscle function occurred within the first 1–2 min after an intense bout of self‐paced, high‐intensity dynamic exercise (Froyd et al. 2013). While significant recovery of markers of neuromuscular function should not be overlooked, the time taken to assess fatigue was consistent between trials and our results further indicate that fatigue resulting from self‐paced exercise was not hypoxia severity‐dependent.

During hypoxic isometric knee extensions, hypocapnia impairs cerebral oxygenation and central drive, but has some protective effects on fatigability in the muscle (Rupp et al. 2015). To which extent CO_2_ breathing (i.e., clamping) is effective in preventing the development of hyperventilation‐induced hypocapnia during intense hypoxic exercise as performed here and what could be the “carry‐over” effects on subsequent performance, physiological responses, and neuromuscular consequences need to be researched. Although standard, our neuromuscular assessment based on MVC of an isolated muscle group (knee extensors) pre‐ versus postcycling might not truly be representative of effective cerebral functioning (and to a lower extent of muscle mechanics) during an actual whole‐body dynamic exercise. Rather, future research would benefit from a combination of methodologies (i.e., perfusion, oxygenation, metabolism, neuronal excitability, and electrical activity) offering complementary insights into the brain in hypoxia (Vergès et al. 2012).

An important consideration when measuring stationary cycling performance using normobaric hypoxic gas mixtures in a laboratory setting is that it does not simulate the terrestrial altitude environment where there is a decrease in air density (i.e., drag forces) due to the decrease in barometric pressure (Peronnet et al. 1991). Interestingly, decreases in time‐trial performance (~8%) and Spo_2_ (~2%) values from normoxia to hypoxia were greater in hypobaric versus normobaric hypoxia for a terrestrial/simulated altitude of 3450 m (Saugy et al. 2016), corresponding to the present more severe hypoxic condition of TT_1_. Our conclusions must therefore remain specific to the context of our study.

## Conclusion

During an initial cycling time‐trial (approximately 16 min), power output was lower and cardiorespiratory load higher in more severe hypoxia, whereas quadriceps muscle activation (twitch interpolation) trend differed between moderate and severe hypoxia. This resulted, however, in the attainment of similar neuromuscular fatigue characteristics at exercise cessation, thus independent of the hypoxic severity. After 22 min of rest, muscle activation returned near baseline, while peripheral function remained depressed. There was no influence of the altitude of a prior time‐trial on performance and associated cardiometabolic responses, with also no additional muscle fatigue development, during a subsequent 6‐min time‐trial performed in moderate hypoxia. We conclude that prior time‐trial performed at higher altitude did not influence further performance and associated cardiometabolic and neuromuscular responses during completion of a subsequent exercise of similar nature in moderate hypoxia.

## Conflict of Interest

The authors have no conflict of interest to disclose.

## References

[phy212804-bib-0001] Allen, D. G. , G. D. Lamb , and H. Westerblad . 2008 Impaired calcium release during fatigue. J. Appl. Physiol. 104:296–305. 17962573 10.1152/japplphysiol.00908.2007

[phy212804-bib-0002] Amann, M. 2011 Central and peripheral fatigue: interaction during cycling exercise in humans. Med. Sci. Sports Exerc. 43:2039–2045. 21502884 10.1249/MSS.0b013e31821f59ab

[phy212804-bib-0003] Amann, M. , and J. A. Dempsey . 2008 Locomotor muscle fatigue modifies central motor drive in healthy humans and imposes a limitation to exercise performance. J. Physiol. 586:161–173. 17962334 10.1113/jphysiol.2007.141838 PMC2375542

[phy212804-bib-0004] Amann, M. , L. M. Romer , D. F. Pegelow , A. J. Jacques , C. J. Hess , and J. A. Dempsey . 2006a Effects of arterial oxygen content on peripheral locomotor muscle fatigue. J. Appl. Physiol. 101:119–127. 16497836 10.1152/japplphysiol.01596.2005

[phy212804-bib-0005] Amann, M. , M. W. Eldridge , A. T. Lovering , M. K. Stickland , D. F. Pegelow , and J. A. Dempsey . 2006b Arterial oxygenation influences central motor output and exercise performance via effects on peripheral locomotor muscle fatigue in humans. J. Physiol. 575:937–952. 16793898 10.1113/jphysiol.2006.113936 PMC1995675

[phy212804-bib-0006] Amann, M. , D. F. Pegelow , A. J. Jacques , and J. A. Dempsey . 2007 Inspiratory muscle work in acute hypoxia influences locomotor muscle fatigue and exercise performance of healthy humans. Am. J. Physiol. Regul. Integr. Comp. Physiol. 293:R2036–R2045. 17715180 10.1152/ajpregu.00442.2007

[phy212804-bib-0007] Chapman, R. F. 2013 The individual response to training and competition at altitude. Br. J. Sports Med. 47:i40–i44. 24282206 10.1136/bjsports-2013-092837 PMC3903142

[phy212804-bib-0008] Christian, R. J. , D. J. Bishop , F. Billaut , and O. Girard . 2014 Peripheral fatigue is not critically regulated during maximal, intermittent, dynamic leg extensions. J. Appl. Physiol. 117:1063–1073. 25213635 10.1152/japplphysiol.00988.2013

[phy212804-bib-0009] Dahlstrom, B. K. , W. R. D. Duff , S. Poloskei , S. Schaerz , T. K. Len , and J. P. Neary . 2013 Neuromuscular changes following simulated high‐intensity cycling performance in moderate hypoxia. J. Exerc. Sci. Fit. 11:78–84.

[phy212804-bib-0010] Dempsey, J. A. , L. Romer , J. Rodman , J. Miller , and C. Smith . 2006 Consequences of exercise‐induced respiratory muscle work. Respir. Physiol. Neurobiol. 151:242–250. 16616716 10.1016/j.resp.2005.12.015

[phy212804-bib-0011] Donnelly, J. , and S. Green . 2013 Effect of hypoxia on the dynamic response of hyperaemia in the contracting human calf muscle. Exp. Physiol. 98:81–93. 22689444 10.1113/expphysiol.2012.066258

[phy212804-bib-0012] Enoka, R. M. , and J. Duchateau . 2016 Translating fatigue to human performance. Med. Sci. Sports Exerc. in press. 10.1249/MSS.0000000000000929 PMC5035715 27015386

[phy212804-bib-0013] Froyd, C. , G. Y. Millet , and T. D. Noakes . 2013 The development of peripheral fatigue and short‐term recovery during self‐paced high‐intensity exercise. J. Physiol. 591:1339–1346. 23230235 10.1113/jphysiol.2012.245316 PMC3607875

[phy212804-bib-0014] Froyd, C. , F. G. Beltrami , G. Y. Millet , and T. D. Noakes . 2016 Central regulation and neuromuscular fatigue during exercise of different durations. Med. Sci. Sports Exerc. in press. 10.1249/MSS.0000000000000867 26741123

[phy212804-bib-0015] Fulco, C. S. , S. F. Lewis , P. N. Frykman , R. Boushel , S. Smith , E. A. Harman , et al. 1996 Muscle fatigue and exhaustion during dynamic leg exercise in normoxia and hypobaric hypoxia. J. Appl. Physiol. 81:1891–1900. 8941506 10.1152/jappl.1996.81.5.1891

[phy212804-bib-0016] Girard, O. , F. Brocherie , J.‐B. Morin , and G. P. Millet . 2015 Neuro‐mechanical determinants of repeated treadmill sprints ‐ Usefulness of an “hypoxic to normoxic recovery” approach. Front. Physiol. 6:260. 26441679 10.3389/fphys.2015.00260 PMC4585155

[phy212804-bib-0017] Goodall, S. , E. Z. Ross , and L. M. Romer . 2010 Effect of graded hypoxia on supraspinal contributions to fatigue with unilateral knee‐extensor contractions. J. Appl. Physiol. 109:1842–1851. 20813979 10.1152/japplphysiol.00458.2010

[phy212804-bib-0018] Harms, C. A. , M. A. Babcock , S. R. McClaran , D. F. Pegelow , G. A. Nickele , W. B. Nelson , et al. 1997 Respiratory muscle work compromises leg blood flow during maximal exercise. J. Appl. Physiol. 82:1573–1583. 9134907 10.1152/jappl.1997.82.5.1573

[phy212804-bib-0019] Harms, C. S. , S. R. McClaran , G. A. Nickele , D. F. Pegelow , W. B. Nelson , and J. A. Dempsey . 2000 Effect of exercise‐induced arterial O_2_ desaturation on VO_2max_ in women. Med. Sci. Sports Exerc. 32:1101–1108. 10862536 10.1097/00005768-200006000-00010

[phy212804-bib-0020] Hogan, M. C. , and H. G. Welch . 1984 Effect of varied lactate levels on bicycle ergometer performance. J. Appl. Physiol. 57:507–513. 6432755 10.1152/jappl.1984.57.2.507

[phy212804-bib-0021] Hureau, T. J. , N. Olivier , G. Y. Millet , O. Meste , and G. M. Blain . 2014 Exercise performance is regulated during repeated sprints to limit the development of peripheral fatigue beyond a critical threshold. Exp. Physiol. 99:951–963. 24728680 10.1113/expphysiol.2014.077974

[phy212804-bib-0022] Karlsson, J. , F. Bonde‐Petersen , J. Henriksson , and H. G. Knuttgen . 1975 Effects of previous exercise with arms or legs on metabolism and performance in exhaustive exercise. J. Appl. Physiol. 38:763–767. 1126883 10.1152/jappl.1975.38.5.763

[phy212804-bib-0023] Millet, G. Y. , D. Aubert , F. B. Favier , T. Busso , and H. Benoît . 2009 Effect of acute hypoxia on central fatigue during repeated isometric leg contractions. Scand. J. Med. Sci. Sports 19:695–702. 18627554 10.1111/j.1600-0838.2008.00823.x

[phy212804-bib-0024] Millet, G. Y. , M. Muthalib , M. Jubeau , P. B. Laursen , and K. Nosaka . 2012 Severe hypoxia affects exercise performance independently of afferent feedback and peripheral fatigue. J. Appl. Physiol. 112:1335–1344. 22323647 10.1152/japplphysiol.00804.2011

[phy212804-bib-0025] Minett, G. M. , and R. Duffield . 2014 Is recovery driven by central or peripheral factors? A role for the brain in recovery following intermittent‐sprint exercise. Front. Physiol. 5:24. 24550837 10.3389/fphys.2014.00024 PMC3909945

[phy212804-bib-0026] Mollard, P. , X. Woorons , M. Letournel , J. Cornolo , C. Lamberto , M. Beaudry , et al. 2007 Role of maximal heart rate and arterial O_2_ saturation on the decrement of VO_2max_ in moderate acute hypoxia in trained and untrained men. Int. J. Sports Med. 28:186–192. 17024632 10.1055/s-2006-924215

[phy212804-bib-0027] Peltonen, J. E. , H. O. Tikkanen , J. J. Ritola , M. Ahotupa , and H. K. Rusko . 2001 Oxygen uptake response during maximal cycling in hyperoxia, normoxia and hypoxia. Aviat. Space Environ. Med. 72:904–911. 11601554

[phy212804-bib-0028] Périard, J. , and S. Racinais . 2016 Performance and pacing during cycle exercise in hyperthermic and hypoxic conditions. Med. Sci. Sports Exerc. 48:845–853. 26656777 10.1249/MSS.0000000000000839

[phy212804-bib-0029] Peronnet, F. , G. Thibault , and D. L. Cousineau . 1991 A theoretical analysis of the effect of altitude on running performance. J. Appl. Physiol. 70:399–404. 2010398 10.1152/jappl.1991.70.1.399

[phy212804-bib-0030] Place, N. , N. A. Maffiuletti , A. Martin , and R. Lepers . 2007 Assessment of the reliability of central and peripheral fatigue after sustained maximal voluntary contraction of the quadriceps muscle. Muscle Nerve 35:486–495. 17221875 10.1002/mus.20714

[phy212804-bib-0031] Romer, L. M. , A. T. Lovering , H. C. Haverkamp , D. F. Pegelow , and J. A. Dempsey . 2006 Effect of inspiratory muscle work on peripheral fatigue of locomotor muscles in healthy humans. J. Physiol. 571:425–439. 16373384 10.1113/jphysiol.2005.099697 PMC1796794

[phy212804-bib-0032] Rupp, T. , T. Le Roux Mallouf , S. Perrey , B. Wuyam , G. Y. Millet , and S. Vergès . 2015 CO_2_ clamping, peripheral and central fatigue during hypoxic knee extensions in men. Med. Sci. Sports Exerc. 47:2513–2524. 26110698 10.1249/MSS.0000000000000724

[phy212804-bib-0033] Saugy, J. , T. Rupp , R. Faiss , A. Lamon , N. Bourdillon , and G. P. Millet . 2016 Cycling time‐trial is more altered in hypobaric than normobaric hypoxia. Med. Sci. Sports Exerc. 48:680–688. 26559447 10.1249/MSS.0000000000000810

[phy212804-bib-0034] Thomas, K. , S. Goodall , M. Stone , G. Howatson , A. St Clair Gibson , and L. Ansley . 2015 Central and peripheral fatigue in male cyclists after 4‐, 20‐, and 40‐km time‐trials. Med. Sci. Sports Exerc. 47:537–546. 25051388 10.1249/MSS.0000000000000448

[phy212804-bib-0035] Torres‐Peralta, R. , J. Losa‐Reyna , M. González‐Izal , I. Perez‐Suarez , J. Calle‐Herrero , M. Izquierdo , et al. 2014 Muscle activation during exercise in severe acute hypoxia: role of absolute and relative intensity. High Alt. Med. Biol. 15:472–482. 25225839 10.1089/ham.2014.1027 PMC4273184

[phy212804-bib-0036] Ventura, N. , H. Hoppeler , R. Seiler , A. Binggeli , P. Mullis , and M. Vogt . 2003 The response of trained athletes to six weeks of endurance training in hypoxia or normoxia. Int. J. Sports Med. 24:166–172. 12740733 10.1055/s-2003-39086

[phy212804-bib-0037] Vergès, S. , N. A. Maffiuletti , H. Kerherve , N. Decorte , B. Wuyam , and G. Y. Millet . 2009 Comparison of electrical and magnetic stimulations to assess quadriceps muscle function. J. Appl. Physiol. 106:701–710. 18756009 10.1152/japplphysiol.01051.2007

[phy212804-bib-0038] Vergès, S. , T. Rupp , M. Jubeau , B. Wuyam , F. Esteve , P. Levy , et al. 2012 Cerebral perturbations during exercise in hypoxia. Am. J. Physiol. Regul. Integr. Comp. Physiol. 302:R903–R916. 22319046 10.1152/ajpregu.00555.2011

[phy212804-bib-0039] Wehrlin, J. P. , and J. Hallen . 2006 Linear decrease in VO_2max_ and performance with increasing altitude in endurance athletes. Eur. J. Appl. Physiol. 96:404–412. 16311764 10.1007/s00421-005-0081-9

